# Evaluation of the Toxicity of 5-Aryl-2-Aminoimidazole-Based Biofilm Inhibitors against Eukaryotic Cell Lines, Bone Cells and the Nematode *Caenorhabditis elegans*

**DOI:** 10.3390/molecules191016707

**Published:** 2014-10-16

**Authors:** Hans Steenackers, Akanksha Dubey, Stijn Robijns, Denis Ermolat’ev, Nicolas Delattin, Barbara Dovgan, Lenart Girandon, Mirjam Fröhlich, Katrijn De Brucker, Bruno P. A. Cammue, Karin Thevissen, Jan Balzarini, Erik V. Van der Eycken, Jozef Vanderleyden

**Affiliations:** 1 Centre of Microbial and Plant Genetics (CMPG), Department of Microbial and Molecular Systems, KU Leuven, Kasteelpark Arenberg 20, Box 2460, B-3001 Leuven, Belgium; 2 Laboratory for Organic & Microwave-Assisted Chemistry (LOMAC), Department of Chemistry, KU Leuven, Celestijnenlaan 200F, B-3001 Leuven, Belgium; 3 Educell, Prevale 9, B-1236 Trzin, Slovenia; 4 Laboratory of Virology and Chemotherapy, Rega Institute for Medical Research, Department of Microbiology and Immunology, KU Leuven, Minderbroedersstraat 10, B-3000 Leuven, Belgium; 5 CELL/TRY Ltd., Levičnikova 34, B-8310 Šentjernej, Slovenia; 6 Department of Biochemistry, Molecular and Structural Biology, Jožef Stefan Institute, Jamova 39, B-1000 Ljubljana, Slovenia; 7 Department of Plant Systems Biology, VIB, Technologiepark 927, 9052 Ghent, Belgium

**Keywords:** 5-aryl-2-aminoimidazole, biofilm inhibitor, toxicity, tumor cell lines, bone cells, *Caenorhabditis elegans*

## Abstract

Previously, we have synthesized several series of compounds based on the 5-aryl-2-aminoimidazole scaffold, which showed a preventive activity against microbial biofilms. We here studied the cytotoxicity of the most active compounds of each series. First, the cytostatic activity was investigated against a number of tumor cell lines (L1210, CEM and HeLa). A subset of monosubstituted 5-aryl-2-aminoimidazoles showed a moderate safety window, with therapeutic indices (TIs) ranging between 3 and 20. Whereas introduction of a (cyclo-)alkyl chain at the *N*1-position strongly reduced the TI, introduction of a (cyclo-)alkyl chain or a triazole moiety at the 2*N*-position increased the TI up to 370. Since a promising application of preventive anti-biofilm agents is their use in anti-biofilm coatings for orthopedic implants, their effects on cell viability and functional behavior of human osteoblasts and bone marrow derived mesenchymal stem cells were tested. The 2*N*-substituted 5-aryl-2-aminoimidazoles consistently showed the lowest toxicity and allowed survival of the bone cells for up to 4 weeks. Moreover they did not negatively affect the osteogenic differentiation potential of the bone cells. Finally, we examined the effect of the compounds on the survival of *Caenorhabditis elegans*, which confirmed the higher safety window of 2*N*-substituted 5-aryl-2-aminoimidazoles.

## 1. Introduction

In the last decades it has become clear that microorganisms predominantly live as surface-associated communities, called biofilms, which are embedded in a self-produced exopolymeric matrix [1,2,3,4]. This is reflected by the observation by the U.S. National Institutes of Health that approximately 80% of all microbial infections are related to biofilms [5,6] and that biofilms are ubiquitous in the environment. Within biofilms microorganisms are generally well-protected against the influence of disinfectants, antibiotics [7] and the host immune system [8], and as a consequence biofilms are extremely difficult to eradicate [9]. As such, they cause major problems in medicine, agriculture, (food) industry and the household environment. Biofilm formation by *Pseudomonas aeruginosa* in the lungs of patients suffering from cystic fibrosis (CF) is one of the best studied examples of biofilm involvement in chronic infections. Because the bacteria assemble in biofilms, this chronic infection is often non-curable and eventually results in the death of CF patients [4,10].

**Figure 1:**
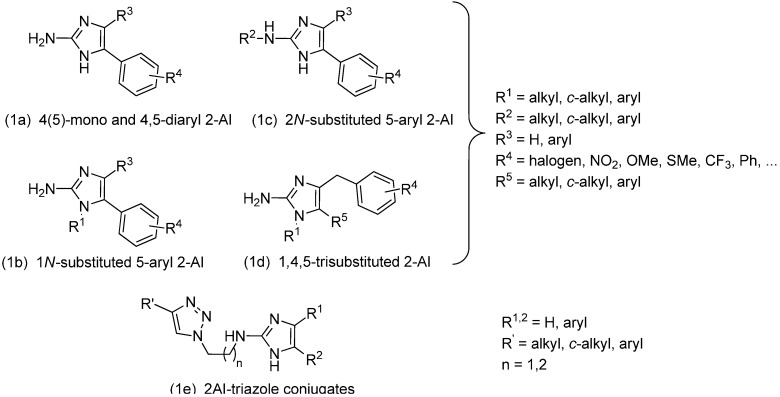
Classes of 5-aryl-substituted 2-AI with anti-biofilm activity reported by our lab [13,14,15,16].

Given the extent of problems caused by biofilms, there has been a significant effort to develop novel anti-biofilm strategies [11,12]. One of the most promising approaches is the exploitation of compounds able to prevent or eradicate biofilms, without affecting the planktonic growth of the microorganisms. These specific anti-biofilm compounds are believed to be less prone to resistance development. Previously, we have developed and reported several series of specific anti-biofilm compounds, based on the 5-aryl-2-aminoimidazole (5-Ar-2AI) scaffold. As illustrated in Figure 1, these series include the mono-substituted 5-Ar-2AIs (**1a**) [13], *N*1-substituted 5-Ar-2AIs (**1b**) [13], 2*N*-substituted 5-Ar-2AIs (**1c**) [14], 4,5-di-substituted 2AIs (**1a**) [13], 1,4,5-trisubstituted 2AIs (**1d**) [15], and 2AI-triazole-conjugates (**1e**) [16]. These compounds show a preventive activity against biofilms of several Gram-positive and Gram-negative bacteria, as well as fungal species, and this in a range of environmental conditions.

**Figure 2:**
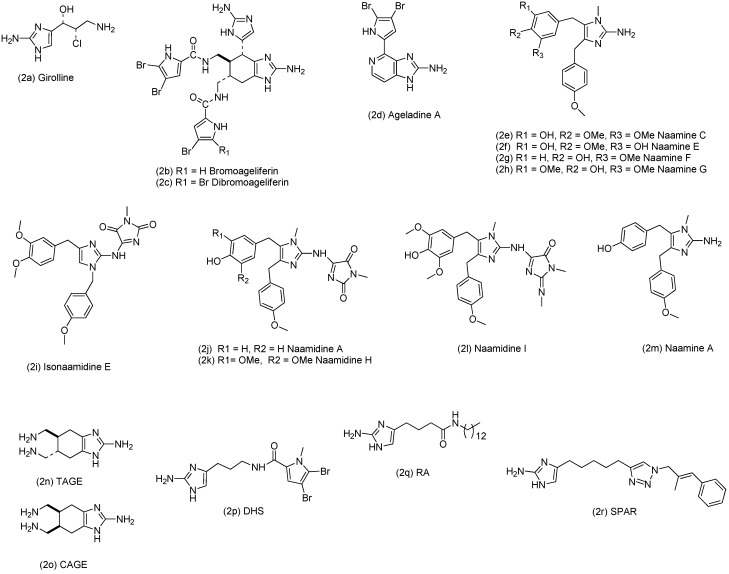
Structures of 2-aminoimidazole (2AI) based compounds, previously tested for cytotoxicity.

Previous studies indicated that the cytotoxicity of 2-aminoimidazole (2AI)-based compounds is strongly dependent on their substitution patterns. Several families of marine sponges produce 2AI-based compounds as a chemical defense mechanism to protect themselves against predators, fight-off competition for space and resources and control surface fouling [17]. Many of these natural 2AIs are cytotoxic [18]. For example, girolline (**2a**, Figure 2), a 2AI isolated from the demosponge *Peudaxinyssa cantharella*, exhibits significant cytotoxicity *in vitro* against several tumor cell lines and *in vivo* against murine-grafted tumors, including P388 and L1210 leukemias, and solid tumors [19,20]. The 2AI-pyrrole alkaloids bromoageliferin (**2b**) and dibromoageliferin **(2c**), isolated from the demosponge *Agelas conifer*, were found to inhibit the calcium entry in PC12 cells [21]. Another 2AI-pyrrole compound, ageladine A (**2d**), isolated from the demosponge *Agelas nakamurai*, was shown to reduce angiogenesis in a model using mouse ES cells, by inhibiting matrix metalloproteinase 2 [22]. Also the 1,4,5-trisubstituted 2AI alkaloids naamine E (**2f**), C (**2e**), F (**2g**) and G (**2h**), the 1,2,4-trisubstituted 2AI isonaamidine E (**2i**) and the tetrasubstituted 2AIs naamidine H (**2k**) and I (**2l**), all isolated from the calcareous sponge *Leucetta chagosensis*, were cytotoxic to a variety of cell lines, including HMO2, HepG2, HeLa and mouse lymphoma cells [23,24,25]. Moreover, the tetrasubstituted 2AI naamidine A (**2j**), also isolated from *L. chagosensis*, was reported to potently inhibit epidermal growth factor-stimulated DNA synthesis in squamous cell tumors and cause A-431 cells to arrest in the G1 phase of the cell cycle [26,27]. While cytotoxicity against tumor cells is desired in anti-cancer applications, it should be avoided in anti-bacterial applications. Therefore, it is interesting to note that several studies indicated that the cytotoxic activity is not inherent to the 2AI-scaffold and can be counteracted by changing the 2AI substitution pattern. Watson *et al.*, for example, found that the known natural product naamine A (**2m**), the C2-dehydrohydantoin deletion analogue of naamidine A, exhibits a markedly decreased ability to inhibit EGFR stimulated DNA synthesis [28]. Using GH4C1 rat pituitary and N2A mouse neuroblastoma cell lines, Melander *et al.* showed that the anti-biofilm compounds TAGE (**2n**) and CAGE (**2o**) lack the cytotoxicity of their parent molecule, bromoageliferin [29]. Moreover, they found that, although the three oroidin-derived anti-biofilm compounds DHS (**2p**), RA (**2q**) and SPAR (**2r**) lack toxicity against *Caenorhabditis elegans*, they show a differential effect on HaCaT keratinocyte cells, again pointing at the importance of the substitution pattern of the 2AI-scaffold [30].

To define which of our 5-Ar-2AI-based anti-biofilm compounds are most promising for further development, we here aimed at the determination of the cytotoxicity of the most active compounds of each subclass. Although mammalian animal models have been a gold standard, they tend to be lengthy and expensive to study. We therefore tested the compounds in a battery of complementary *in vitro* cellular assays. To get a first idea of their cytotoxicity, the cytostatic activity of the compounds was tested against a number of commonly used tumor cell lines, *i.e.*, murine leukemia L1210, human lymphocyte CEM, and human cervix carcinoma HeLa [31,32,33]. Cytostatic activity against multiple tumor cell lines can provide an indication of basal toxicity on fast growing tissue cells (e.g., mucosa, bone marrow, …) [34]. A promising application of specific, preventive anti-biofilm agents is their use in anti-biofilm coatings for implants, such as orthopedic implants. Since it is of great importance that the anti-biofilm compounds not be cytotoxic for bone tissue and do not negatively affect the osseointegrative potential of the implant, their effects on cell viability and functional behavior (differentiation potential) of two of the most relevant cell types represented in bone tissue (human osteoblasts and bone marrow derived mesenchymal stem cells) were also tested. Finally, we examined the effect of the compounds on the survival of *C. elegans*, a nonparasitic nematode species, which has become one of the most extensively studied model organisms. *C. elegans* is a valuable toxicity model since there is increasing evidence that results obtained with *C. elegans* are predictive of outcomes in higher eukaryotes, both at the level of genetic and physiological similarity and at the level of actual toxicity data [35,36]. Indeed, many of the basic physiological processes and stress responses that are observed in higher organisms (e.g., humans) are conserved in *C. elegans* [37]. From these initial studies, promising subsets of compounds can be further subjected to more rigorous investigations.

## 2. Results and Discussion

### 2.1. Cytostatic Activity against Tumor Cell Lines

To get a first idea of their cytotoxicity, a selection of the most active compounds of each subclass of 5-Ar-2AIs was investigated for cytostatic activity against murine leukemia (L1210), human T-lymphocyte (CEM) and human cervix carcinoma (HeLa) cell lines. The IC_50_ was defined as the compound concentration required to inhibit cell proliferation by 50%. The therapeutic index (TI) was calculated as the ratio of the compound concentration producing toxicity (IC_50_) to the concentration needed to exert the desired ‘therapeutic’ effect on biofilms of *Salmonella* and *Pseudomonas*, indicated by the BIC_50_ (dose needed to prevent biofilm formation by 50%). The higher the therapeutic index, the broader the safety window of the compound.

As shown in Table 1, the monosubstituted 5-Ar-2AIs have cytostatic IC_50_ values for all cell lines in the range of 15-150 µM. Comparison with the BIC_50_ for *Salmonella* biofilm inhibition indicates a reasonable safety window for compounds **2** and **4** (bearing resp. a chlorine and a nitro group at the 5-aryl-ring), with TIs ranging between 3 and 6 for all cell lines, while compounds **1**, **3** and **5** are toxic at concentrations overlapping with the *Salmonella* biofilm inhibitory concentrations [13]. In comparison with the BIC_50_ for *Pseudomonas* biofilm inhibition, compound **2** shows an interesting safety window (with TIs between 14 and 22), while compounds **3**–**5** have a moderate safety window (with TIs between 1.5 and 7) and compound **1** is toxic at the biofilm inhibitory concentrations.

We previously reported that introduction of an intermediate length *n*-alkyl or cycloalkyl chain at the *N*1-position of the 5-Ar-2AIs strongly enhances their anti-biofilm activity [13]. However, as indicated in Table 1, these modifications also strongly increase the cytotoxicity of the compounds. Indeed, all the *N*1-substituted 5-Ar-2AIs tested have IC_50_ values in the range of 3-9 µM. As a result these compounds generally have TI’s lower than 1, both with respect to *Salmonella* and *Pseudomonas* biofilm inhibition. Exceptions are compound **10** (*N*1-octyl-5-Ph-2AI), which has a moderate safety window with regard to *Salmonella* biofilm inhibition (TI’s between 2 and 2.5), and compounds **13** (*N*1-cyclo-octyl-5-Ph-2AI) and **15** (*N*1-octyl-5-[4-SMePh]-2AI), which have a moderate safety window with regard to *Pseudomonas* biofilm inhibition (TIs between 1.5 and 2.5) [13].

**Table 1:** Cytostatic activity of 5-Ar-2AI subclasses against tumor cell lines, toxicity against *C. elegans* and anti-biofilm activity against bacterial strains. 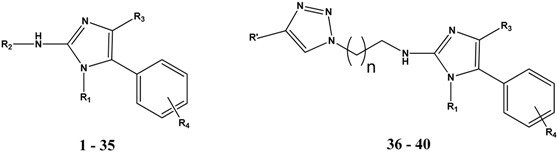

					IC_50_^a^ (µM)				BIC_50_^b^ (µM)		TI ^c^*S.* Typhimurium				TI *P. aeruginosa*				*C. elegans*
n°	R4	R1	R2	R3	L1210	CEM	HeLa		*S*. Typhimurium	*P. aeruginosa*	L1210	CEM	HeLa		L1210	CEM	HeLa		% survival at 25 µM ^e^
monosubstituted																			
**1**	H	H	H	H	62.9	94.3	144.6		130.2	72.6	0.5	0.7	1.1		0.9	1.3	2.0		89.8
**2**	4-Cl	H	H	H	77.5	49.6	72.3		16	3.5	4.8	3.1	4.5		22.1	14.2	20.7		84.9
**3**	4-Ph	H	H	H	37.4	13.6	34.0		17.3	8.6	2.2	0.8	2.0		4.3	1.6	4.0		nd
**4**	4-NO_2_	H	H	H	83.3	63.7	107.7		17.6	34.5	4.7	3.6	6.1		2.4	1.8	3.1		97.3
**5**	4-Br	H	H	H	10.1	13.9	21.8		47.9	3.2	0.2	0.3	0.5		3.2	4.3	6.8		90.6
N1-substituted																			
**6**	H	Oct	H	H	3.2	467.9	3.0		11.9	18.5	0.3	39.3	0.3		0.2	25.3	0.2		92.5
**7**	4-Cl	Oct	H	H	3.9	356.8	4.5		5.9	4.0	0.7	60.5	0.8		1.0	89.2	1.1		86.6
**8**	4-Cl	Trd	H	H	7.2	6.4	4.5		19.5	33.9	0.4	0.3	0.2		0.2	0.2	0.1		nd
**9**	4-Ph	Hept	H	H	7.5	8.1	8.7		9.3	25.2	0.8	0.9	0.9		0.3	0.3	0.3		nd
**10**	4-Ph	Oct	H	H	7.8	8.3	6.6		3.3	8.9	2.4	2.5	2.0		0.9	0.9	0.7		nd
**11**	4-Ph	c-Pen	H	H	4.3	2.9	8.2		7.5	353.1	0.6	0.4	1.1		0.01	0.01	0.02		nd
**12**	4-Ph	c-Hept	H	H	8.1	5.4	6.6		12.5	6.6	0.6	0.4	0.5		1.2	0.8	1.0		nd
**13**	4-Ph	c-Oct	H	H	6.9	4.9	6.9		22.9	3.2	0.3	0.2	0.3		2.2	1.5	2.2		nd
**14**	4-F	Oct	H	H	3.5	7.3	8.6		10.8	4.9	0.3	0.7	0.8		0.7	1.5	1.8		nd
**15**	4-SMe	Oct	H	H	4.1	7.2	6.3		6.7	2.8	0.6	1.1	0.9		1.5	2.6	2.3		nd
**16**	3-Br	Oct	H	H	6.9	9.7	8.0		11.2	40.6	0.6	0.9	0.7		0.2	0.2	0.2		nd
**17**	3,4-diCl	m-MeOPhenethyl	H	H	6.9	8.6	6.6		>400	nd ^d^	<0.02	<0.02	<0.02		nd	nd	nd		nd
2*N*-substituted																			
**18**	H	H	Bu	H	46.4	667.4	19.5		25.3	31.8	1.8	26.4	0.8		1.5	21.0	0.6		nd
**19**	H	H	i-Bu	H	83.6	382.7	91.4		4.9	1.2	17.1	78.1	18.7		69.7	318.9	76.2		nd
**20**	H	H	c-Pen	H	27.3	293.3	27.3		52.9	33.8	0.5	5.5	0.5		0.8	8.7	0.8		98.1
**21**	4-Cl	H	Bu	H	40.0	374.6	34.7		nd	nd	nd	nd	nd		nd	nd	nd		nd
**22**	4-Cl	H	i-Bu	H	336.3	297.3	171.5		2	0.9	168.2	148.7	85.8		373.7	330.3	190.6		97.6
**23**	4-Cl	H	Pen	H	22.0	322.9	19.8		>400	6.3	<0.05	<0.8	<0.05		3.5	51.3	3.1		nd
**24**	4-Cl	H	c-Pen	H	35.5	591.0	28.8		4.4	13.5	8.1	134.3	6.5		2.6	43.8	2.1		100.1
**25**	4-Br	H	Bu	H	125.8	493.0	50.7		7.1	9.8	17.7	69.4	7.1		12.8	50.3	5.2		102.4
**26**	4-Br	H	i-Bu	H	31.7	306.0	22.6		2.9	1.2	10.9	105.5	7.8		26.4	255.0	18.8		nd
**27**	4-Br	H	Pen	H	14.9	375.2	16.3		3.1	10.2	4.8	121.0	5.2		1.5	36.8	1.6		nd
**28**	4-Br	H	c-Pen	H	32.7	673.6	32.7		12.1	7.2	2.7	55.7	2.7		4.5	93.6	4.5		nd
**29**	3,4-diCl	H	c-Pen	H	76.1	155.9	79.8		5.7	7.9	13.4	27.4	14.0		9.6	19.7	10.1		nd
4,5-disubstituted																			
**30**	H	H	H	4-OMePh	64.1	52.8	45.2		77.1	nd	0.8	0.7	0.6		nd	nd	nd		nd
**31**	H	H	H	4-ClPh	59.3	48.2	40.8		46.9	nd	1.3	1.0	0.9		nd	nd	nd		nd
**32**	4-Cl	H	H	4-MePh	12.3	10.2	9.5		12.9	nd	1.0	0.8	0.7		nd	nd	nd		nd
**33**	4-Cl	H	H	4-CF_3_Ph	41.5	35.5	26.1		10.8	nd	3.8	3.3	2.4		nd	nd	nd		nd
**34**	4-F	H	H	amidophenyl	74.2	74.2	77.6		182.1	nd	0.4	0.4	0.4		nd	nd	nd		nd
1,4,5-trisubstituted																			
**35**	4-Me	Ben	H	p-PenOBn	8.6	8.4	14.8		10.3	27.4	0.8	0.8	1.4		0.3	0.3	0.5		nd
2AI-triazole conjugates																			
**36**	H	H	n = 2, R'= Ph	H	221.0	452.6	72.9		36.5	>400	6.1	12.4	2.0		<0.6	<1.1	<0.2		87.7
**37**	H	H	n = 3, R' = Ph	H	217.8	459.2	106.7		40.0	19.0	5.4	11.5	2.7		11.5	24.2	5.6		86.4
**38**	4-OMe	H	n = 2, R' = 4-BrPh	H	18.4	596.6	16.8		91.2	42.7	0.2	6.5	0.2		nd	nd	nd		nd
**39**	4-Br	H	n = 2, R' = c-Hex	H	20.7	396.0	22.5		8.4	12.5	2.5	47.1	2.7		1.7	31.7	1.8		99.3
**40**	4-Br	H	n = 2, R' = c-Pr	H	43.9	33.6	67.1		2.0	71.6	21.9	16.8	33.6		0.6	0.5	0.9		82.6

^a^ IC_50_: compound concentration required to inhibit cell proliferation by 50%. The values represent the means of three repeats. Standard deviations are provided in Table S1; ^b^ BIC_50_: compound concentration required to prevent biofilm formation (at 25 °C) by 50%, as previously reported [13,14,15,16]. 95% confidence intervals are provided in Table S1; ^c^ TI: the ratio of the compound concentration producing toxicity (IC_50_) to the dose needed to exert the desired ‘therapeutic’ effect on biofilms (BIC_50_); ^d^ nd: not determined; ^e^ The % survival of the worms in the presence of anti-biofilm compounds was calculated after 7 days relative to their viability at day 0. The values represent the means of least two repeats. Standard deviations are provided in Table S1.

**Figure 3:**
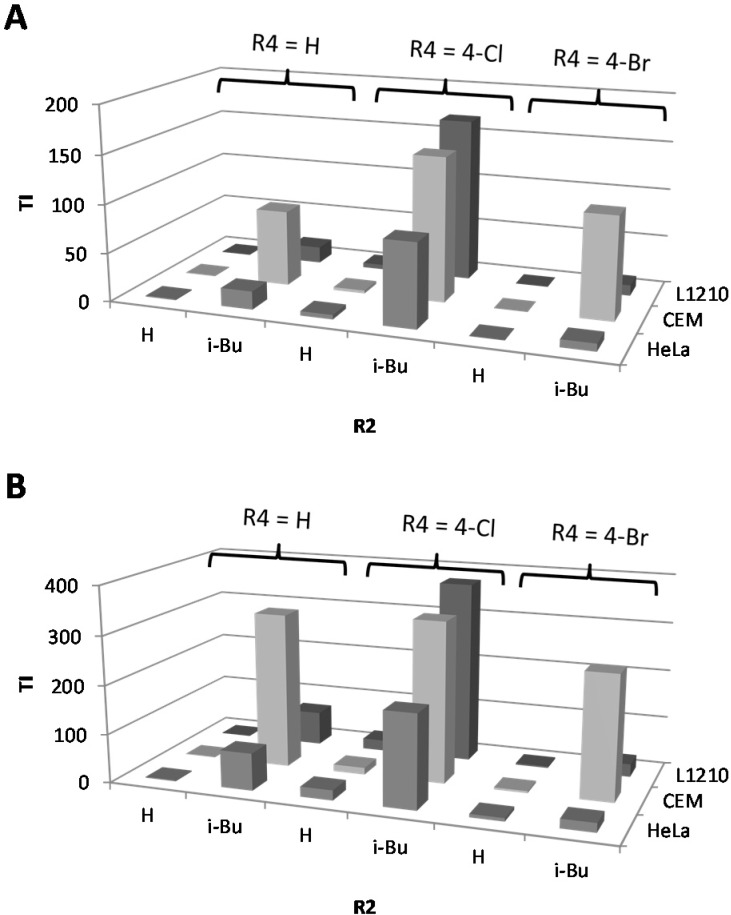
Effect of introduction of an iso-butyl chain at the 2*N*-position (R2) of 5-Ar-2AIs, bearing H, Cl or Br at the para-position of the 5-phenylring (R4), on the therapeutic index (TI) with respect to *Salmonella* Typhimurium biofim inhibition (**A**) and *Pesudomonas aeruginosa* biofilm inhibition (**B**).

**Figure 4:**
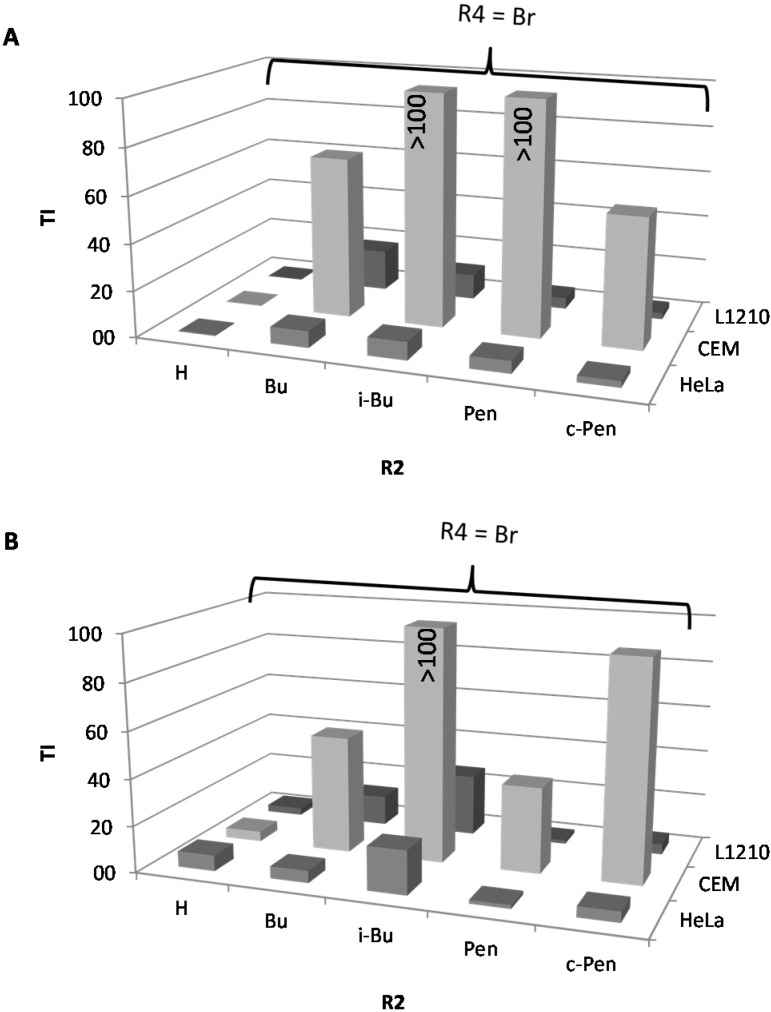
Effect of introduction of an intermediate length (cyclo-)alkyl chain (Bu, *i*-Bu, Pen, c-Pen) at the 2*N*-position of 5-(*p*-bromophenyl)-2-aminoimidazole (compound **5**) on the therapeutic index (TI) with respect to *Salmonella* Typhimurium biofilm inhibition (**A**) and *Pseudomonas aeruginosa* biofilm inhibition (**B**).

We also reported that introduction of an intermediate length alkyl chain (*n*-butyl, *iso*-butyl, cyclo-butyl, *n*-pentyl or cyclopentyl) at the 2*N*-position of the 5-Ar-2AIs can strongly increase their anti-biofilm activity [14]. Interestingly, these 2*N*-substituted 5-Ar-2AIs generally do not show a markedly increased cytotoxicity as compared to the mono-substituted 5-Ar-2AIs. On the contrary, the cytotoxicity is often even reduced, with IC_50_ values ranging between 15 and 600 µM. As a result the safety window of many of these compounds is strongly broadened. More specifically, all tested 5-Ar-2AIs substituted with an *iso*-butyl group at the 2*N*-position have high TIs both with respect to *Salmonella* and *Pseudomonas* biofilm inhibition, irrespective of the nature of the R4 substituent (Figure 3). Especially compound **22**, bearing an *iso*-butyl substituent at the 2*N*-position and *p*-chlorophenyl at the 5-position, has a very broad safety window with TIs in the range of 85 to 380. Also, all tested compounds derived from compound **5** (with *p*-bromo as R4 group) by introduction of an intermediate length alkyl chain at the 2*N*-position (*i.e.*, compounds **25**–**28**) have increased TIs (far higher than 10) with respect to *Salmonella* and/or *Pseudomonas* biofilm inhibition (Figure 4). Except for compounds **20** and **23**, which have TIs below 1, all other compounds tested show higher safety windows. This points to the potential of the *2N*-alkyl-substituted 5-Ar-2AIs to be further explored and developed into safe anti-biofilm agents. It should be noticed that CEM cell cultures seem to be less sensitive to the antiproliferative activity of the 2*N*-substituted and 2AI-triazole conjugate derivatives, but not the other compounds, compared to L1210 and HeLa cell cultures. The molecular basis of this observation is currently unclear.

As previously reported, the 4,5-di-substituted 5-Ar-2AIs are active against biofilms at similar doses as the mono-substituted 5-Ar-2AIs [13]. As indicated in Table 1, also the cytotoxic activity occurs at similar concentrations, with IC_50_ values between 10 and 80 µM, resulting in similar TIs. Only compound **33**, 4,5-substituted with a *p*-chlorophenyl and a *p*-trifluoromethylphenyl group, shows a moderate safety window with respect to *Salmonella* biofilm inhibition, with TIs between 2.4 and 3.8.

The 1,4,5-trisubstituted compound **35** has therapeutic indices below 1 with respect to biofilm inhibition of both bacterial species, again demonstrating that introduction of an alkyl substituent at the *N*1-position does not improve the safety window [15].

Finally, a number of the most active 2AI-triazole conjugates were tested for cytotoxicity [16]. In these compounds a triazole moiety is coupled to the 2*N*-position of the 2AI-ring via an ethyl or propyl linkage. Although to a lesser extent than for the 2*N*-alkyl-substituted 2AIs, also the 2AI-triazole conjugates generally show an increased safety window with respect to *Salmonella* and/or *Pseudomonas* biofilm inhibition, as compared to the 2*N*-unsubstituted compounds, with TI values ranging between 2 and 50 for compounds **36**, **37**, **39** and **40**. Especially compound **40** shows a promising safety window with regard to *Salmonella* biofilm inhibition with TI values higher than 16 for all cell lines [16]. Triazole click chemistry provides an easy way to immobilize 2AIs to a surface, generating a triazole moiety between the 2AI and the surface. The present data indicate that this is not only an easy but also a safe method for 2AI immobilization.

### 2.2. Effects on Viability and Functional Behavior of Bone Cells

A promising application of specific, preventive anti-biofilm agents is their use in anti-biofilm coatings for implants, such as orthopedic implants. The osseointegrative properties of the implant are crucial for the long-term functional bone regeneration. Therefore it is of great importance that the applied anti-biofilm agents are not cytotoxic for osteoblasts (OB) and mesenchymal stem cells (MSC) and do not negatively affect the osseointegrative potential of the implant [38]. Therefore, we tested these features for a selection of three compounds displaying high TI’s, namely **22**, **25** and **40**, and compound **7**, which was more toxic against the tumor cell lines.

**Figure 5:**
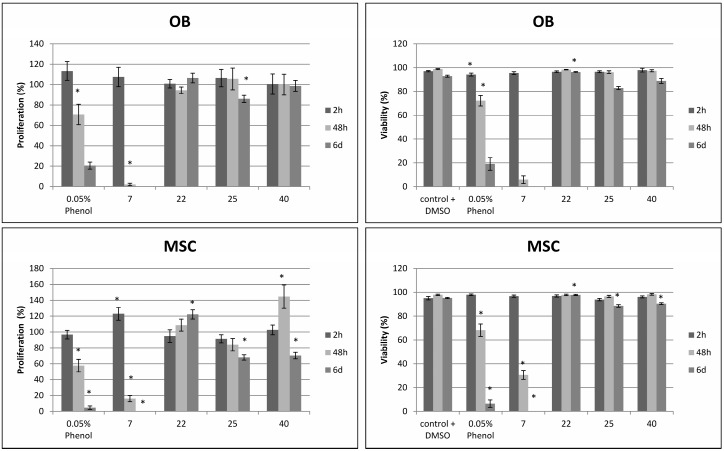
Effect of selected compounds (12.5 µM) on the proliferation and viability of osteoblasts (OB) and mesenchymal stem cells (MSC) after 2 h, 48 h and 6 days of exposure, as determined by trypan blue staining. Negative controls were cell culture medium and cell culture medium with 0.5% DMSO solvent background, positive control was 0.05% phenol to show a cytotoxic effect. % Proliferation is defined as: (total viable cells in treated sample/total viable cells in solvent control) × 100. % Viability is defined as: total viable cells (unstained)/total cells (stained + unstained) × 100. Bars and error bars represent resp. means and standard errors of 8 repeats. Significant differences (*p* < 0.05) with the negative solvent control are indicated with an asterix (*).

Firstly, the effect of compounds **7**, **22**, **25** and **40** was tested on the proliferation (i.e., percentage of viable cells in treated sample compared to viable cells in solvent control) and viability (i.e., percentage of viable cells in treated sample compared to total number (viable and non-viable) of cells in treated sample) of OB and MSC in function of time. For each compound a dose of 12.5 µM was used, which is well above the BIC_50_ value of all compounds for *Salmonella* Typhimurium biofilm inhibition and *P. aeruginosa* biofilm inhibition (except for compound **40**, which has a *P. aeruginosa* BIC_50_ of 72 µM). Direct measurement of cell viability by trypan blue staining indicated that, after 48h exposure to 12.5 µM of compound **7**, the proliferation and viability of both cell types was strongly reduced as compared to the solvent control (>70% reduction, Figure 5). In contrast, compounds **22**, **25** and **40** (12.5 µM) do not or only slightly reduce the proliferation and viability of either of the two cell types after 48h and after 6 days of exposure. Compounds **22**, **25** and **40** even allow survival of MSC and OB after 4 weeks of exposure. Consistently, MTT staining indicated a strongly reduced metabolic activity for both cell types (>80% as compared to the untreated controls) after 6 days exposure to 12.5 µM of compound **7**, while compounds **22**, **25** and **40** (12.5 µM, 6 days exposure) even slightly enhance the metabolic activity (results not shown). These results are in line with the results with the tumor cell lines and emphasize the potential of the 2*N*-substituted compounds **22**, **25**, **40** to be applied in anti-biofilm coatings for orthopedic implants.

**Figure 6:**
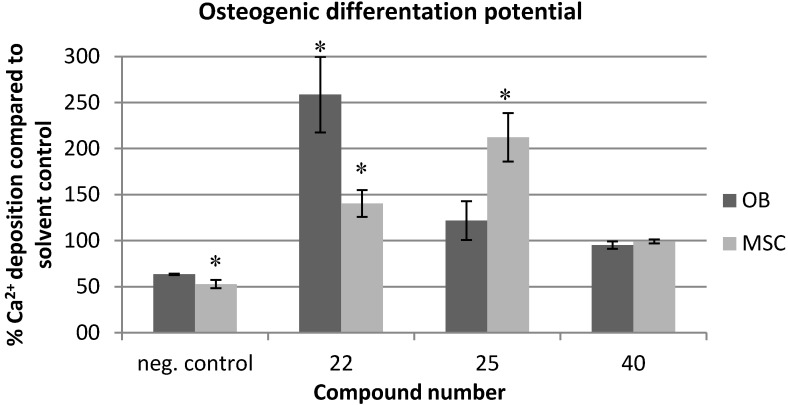
Effect of selected compounds (12.5 µM) on the osteogenic differentiation potential of osteoblasts (OB) and mesenchymal stem cells (MSC) after resp. 3 and 5 weeks of exposure, as determined by measuring the amount of calcium deposition compared to the solvent control. Bars and error bars represent resp. means and standard errors of at least four repeats. The negative control contains no osteogenic supplements. The solvent control contains osteogenic supplements and a 0.5% DMSO background. Significant differences (*p* < 0.05) with the solvent control are indicated with an asterix (*).

Next, the effect of compounds **22**, **25** and **40**, which showed the lowest toxicity against the bone cells and allowed survival of MSC and OB cells for more than 3 weeks, was tested with respect to the osteogenic differentiation potential of MSC and OB, as those two cell types are responsible for the production of new bone matrix within bone tissue. Calcium deposition was chosen as an indicator of the osteogenic phenotype, as it is the final and functional marker of osteoblast differentiation. As shown in Figure 6, none of the compounds does negatively affect the calcium deposition of either of the two cell types at 12.5 µM.

Interestingly, compound **22** even significantly (*p* < 0.05) induces the calcium deposition of both cell types, while compound **25** significantly induces the calcium deposition of MSC. This suggests that anti-biofilm coatings with these compounds might possibly even stimulate the osseointegrative potential of orthopedic implants.

### 2.3. Evaluation of the Toxicity against C. elegans

**Figure 7:**
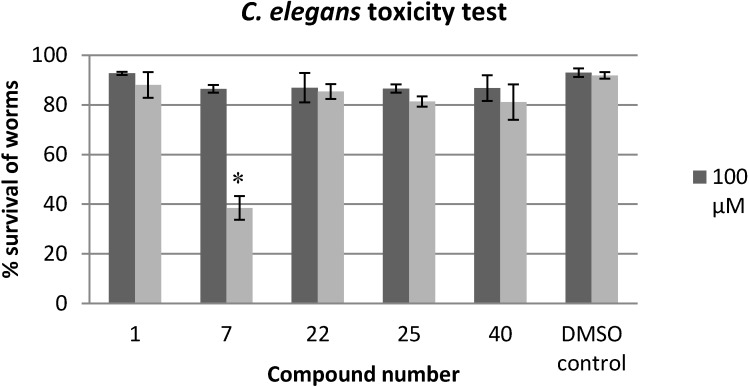
Effect of selected compounds (100 and 200 µM) on the survival of *C. elegans*, after 7 days of exposure. The % survival of the worms in the presence or absence (DMSO background) of anti-biofilm compounds was calculated after 7 days relative to their viability at day 0. Bars and error bars represent resp. means and standard errors of four repeats. Significant differences (*p* < 0.05) with the DMSO control are indicated with an asterix (*).

The multicellular nematode worm *C. elegans* is regarded as one of the best multicellular models to understand the biology of all animals, including humans [39]. Therefore, we tested the effect of a variety of monosubstituted, *N*1-substituted and 2*N*-substituted 5-Ar-2AIs, as well as 2AI-triazole conjugates, on the survival of *C. elegans* (Table 1). Although the tested compounds include both compounds with a low cytotoxicity against the tumor cell lines (e.a. **22** and **25**) and a higher cytotoxicity (e.a. **6** and **7**), all compounds allow a survival of more than 80% of the *C. elegans* nematodes after 7 days exposure to a dose of 25 µM. As such, the tumor cell lines seem to be more sensitive to the 2AIs as compared to the multicellular *C. elegans* model. To further evaluate their safety window a selected number of compounds (**1**, **7**, **22**, **25** and **40)** was tested at higher concentrations. As shown in Figure 7, none of the compounds significantly affect *C. elegans* survival at 100 µM. At a concentration of 200 µM, the mono-substituted 5-Ar-2AI **1** and the 2*N*-substituted 5-Ar-2AIs **22**, **25** and **40** do not significantly affect survival, while the *N*1-substituted 5-Ar-2AI **7** inhibits survival by more than 50%. This again points at the higher toxicity of the *N*1-substituted compounds.

## 3. Experimental Section

### 3.1. Compound Preparation

All compounds were synthesized according to our previous reported protocols [13,14,15,16], after which stock solutions in DMSO (20, 25 or 50 mM) were prepared.

### 3.2. Cytostatic Activity against Tumor Cell Lines

Murine leukemia (L1210), human T-lymphocyte (CEM) and human cervix carcinoma (HeLa) were suspended at 300,000–500,000 cells/mL of culture medium, and 100 µL of a cell suspension was added to 100 µL of an appropriate dilution of the test compounds in 200 µL-wells of 96-well microtiter plates. After incubation at 37 °C for two (L1210), three (CEM) or four (HeLa) days, the cell number was determined using a Coulter counter. The IC_50_ was defined as the compound concentration required to inhibit cell proliferation by 50%.

### 3.3. Cytotoxicity against Osteoblasts and Mesenchymal Stem Cells

The cytotoxicity of the compounds was measured on cell cultures of two cell types represented in bone tissue, namely human osteoblasts (OB) and human bone marrow-derived mesenchymal stem cells (MSC). These tests were conducted according to ISO 10993-5 cytotoxicity standard. The cells were seeded in a 96-well plate at a density of 5000 cells/cm^2^ in cell culture media (Advanced DMEM, 10% FBS, 1× GlutaMAX, 0.05 mg/mL gentamicin) and allowed to attach overnight. The next day the test compounds (12.5 μM) and controls were added to the cells (negative controls were cell culture medium and cell culture medium with 0.5% DMSO background, positive control was 0.05% phenol to show a cytotoxic effect) and incubated for 2h, 48h and 6 days and at each time-point the numbers of viable and dead cells were determined by trypan blue staining, and the metabolic activity was determined with MTT staining.

Trypan blue staining: the medium was removed from the wells, and 1/3 trypan blue in DMEM medium was added to the cells, incubated for 3 min, after which trypan blue was removed and DMEM medium was added to the wells. In each of four wells, two microscopy fields were counted for viable (transparent) and dead (blue) cells.

MTT: the medium was removed from the wells and 100 μL of medium, supplemented with 10% serum and 0.5 mg/mL MTT was added to the cells. The cells were incubated overnight at 37 °C and 5% CO_2_. The next day the medium with MTT was removed and 100 μL acidic isopropanol was added. The cells were then centrifuged at 2300 g and 50 μL of the supernatant was transferred to a new 96-well plate. The absorbance was measured at 570 nm and the background was measured at 660 nm. Four wells per condition were examined.

### 3.4. Calcium Assay for Determination of Osteogenic Differentiation Potential

MSC and OB were cultured in osteogenic medium for 3 and 5 weeks, respectively. The experimental groups included a positive solvent control (osteogenic medium with 0.5% DMSO background), a negative control (medium without osteogenic supplements) and treated samples (osteogenic medium, 0.5% DMSO background and 12.5 µM of test compound). Cell cultures were extracted by 5% trichloroacetic acid (500 μL per sample). *O*-Cresolphtalein complex was added (Calcium CPC LiquiColor Test^®^; Stanbio Laboratory, Boerne, TX, USA), and the calcium content was determined spectrophotometrically at 550 nm.

To determine the DNA content, cells were washed with PBS, and 200 μL of digestion buffer (10 mM Tris, 1 mM EDTA, 0.1% Triton X-100, 0.1 mg/mL proteinase K) was added. Samples were incubated in digestion buffer overnight at 56 °C. The supernatants were drawn off and PicoGreen^®^ dye (Molecular Probes, Eugene, OR, USA) was added to the samples in 1:1 ratio and read in a fluorescent plate reader (excitation 485 nm and emission 528 nm). DNA values were used to normalize the calcium content. Four wells per condition were examined, and two samples from each well were taken for each assay.

### 3.5. C. elegans Toxicity Test

Eggs of a double mutant (glp-4Dsek-1D) of *C. elegans* were grown on NGM/OP50 agar plates (NGM agar plates on the surface inoculated with 100 µL of an overnight culture of OP50 *Escherichia coli* and stored at 4 °C until use) at 25 °C until all nematodes had reached the L4 stage. Worms were collected and washed with M9 buffer (3 g/L KH_2_PO_4_, 6 g/L Na_2_HPO_4_, 5 g/L NaCl, 1 mM MgSO_4_). For toxicity testing, 40 to 50 worms were suspended in 250 μL M9 (supplemented with 10 µg/mL cholesterol, 75 µg/mL ampicillin and 100 µg/mL kanamycin) buffer in each well of 24-well microtiter plates, in the presence or absence (0.5% DMSO control) of 25, 100 or 200 µM of the test compounds and grown for 7 days at 25 °C. The percentage survival of the worms in the presence or absence of anti-biofilm compounds was calculated after 7 days relative to their viability at day 0.

## 4. Conclusions

To define which of our 5-aryl-2-aminoimidazole-based anti-biofilm compounds are the most promising for further development, we studied the cytotoxicity of the most active compounds of each subclass. With regard to the cytostatic effect on the tumor cell lines (L1210, CEM, and HeLa) the aminoimidazoles substituted with a (cyclo-)alkyl chain or a triazole moiety at the 2*N*-position showed the broadest safety window with TI’s up to 370. Consistently, the 2*N*-substituted 2-aminoimidazoles did not affect cell viability and functional behavior of bone cells (osteoblasts and mesenchymal stem cells) nor the viability of the model organism *C. elegans*. Therefore, from these initial studies, 2*N*-substituted 5-aryl-2-aminoimidazoles seem to be most promising as anti-biofilm agents.

## Supplementary Materials

Table S1 showing the cytostatic activity of 5-Ar-2AI subclasses against tumor cell lines, toxicity against *C. elegans* and anti-biofilm activity against bacterial strains is available at http://www.mdpi.com/1420-3049/19/10/16707/s1.

## Supplementary Files

Supplementary File 1
