# Supplementary material for: Evaluation of the Toxicity of 5-Aryl-2-Aminoimidazole-Based Biofilm Inhibitors against Eukaryotic Cell Lines, Bone Cells and the Nematode Caenorhabditis elegans
Source: Molecules. 2014 Oct 16;19(10):16707–23. doi: 10.3390/molecules191016707 (PMC6271933; doi:10.3390/molecules191016707)
Supplement: Supplementary File 1 [file molecules-19-16707-s001.pdf]

Supplementary Materials

Table S1. Cytostatic activity of 5-Ar-2AI subclasses against tumor cell lines, toxicity against *C. elegans* and anti-biofilm activity against bacterial strains.

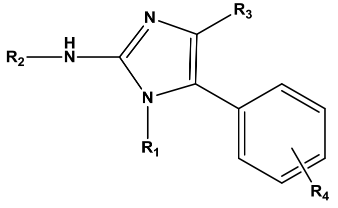

1 - 35

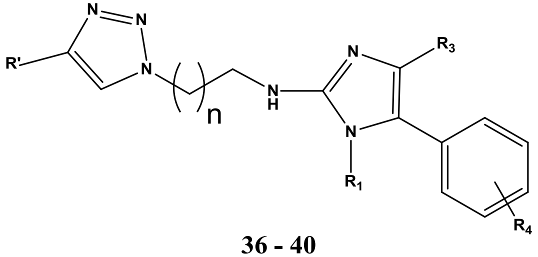

36 - 40

					IC_{50} [a] (µM)			SD for IC_{50} (µM)			BIC_{50} [b] (µM)		95% Confidence Interval for BIC_{50}		*C.elegans*	
n°	R4	R1	R2	R3	L1210	CEM	HeLa	L1210	CEM	HeLa	*S.*Typhimurium	*P. aeruginosa*	*S.* Typhimurium	*P. aeruginosa*	% Survival at 25 µM [d]	SD for % Survival at 25 µM
		monosubstituted														
**1**	H	H	H	H	62.9	94.3	144.6	31.4	75.4	31.4	130.2	72.6	112.6–150.7	38.7–136.2	89.8	8.2
**2**	4-Cl	H	H	H	77.5	49.6	72.3	25.8	8.3	5.2	16	3.5	14.3–17.9	2.3–5.2	84.9	6.0
**3**	4-Ph	H	H	H	37.4	13.6	34.0	25.9	4.3	1.3	17.3	8.6	15.9–18.8	4.8–15.6	nd	
**4**	4-NO_2	H	H	H	83.3	63.7	107.7	4.9	14.7	14.7	17.6	34.5	15.0–20.5	20.4–58.8	97.3	7.3
**5**	4-Br	H	H	H	10.1	13.9	21.8	2.5	9.7	1.7	47.9	3.2	36.6–62.8	2.3–4.5	90.6	10.8
		N1-substituted														
**6**	H	Oct	H	H	3.2	467.9	3.0	1.1	2.2	1.1	11.9	18.5	10.2–13.8	8.4–40.7	92.5	19.8
**7**	4-Cl	Oct	H	H	3.9	356.8	4.5	1.3	0.7	1.0	5.9	4.0		3.0–5.2	86.6	14.2
**8**	4-Cl	Trd	H	H	7.2	6.4	4.5	0.3	1.9	3.5	19.5	33.9	11.1–34.5	15.0–76.4	nd	
**9**	4-Ph	Hept	H	H	7.5	8.1	8.7	0.3	1.2	1.2	9.3	25.2	6.9–12.4	15.6–40.9	nd	
**10**	4-Ph	Oct	H	H	7.8	8.3	6.6	1.2	0.0	1.4	3.3	8.9	1.2–9.0	3.5–22.5	nd	
**11**	4-Ph	c-Pen	H	H	4.3	2.9	8.2	2.3	1.4	1.0	7.5	353.1	5.5–10.3	151.2–824.6	nd	
**12**	4-Ph	c-Hept	H	H	8.1	5.4	6.6	1.5	4.2	3.9	~12.5	6.6		2.9–14.7	nd	
**13**	4-Ph	c-Oct	H	H	6.9	4.9	6.9	6.8	88.4	10.2	~22.9	3.2		1.5–6.7	nd	
**14**	4-F	Oct	H	H	3.5	7.3	8.6	0.0	2.4	1.0	~10.8	4.9		3.8–6.2	nd	
**15**	4-SMe	Oct	H	H	4.1	7.2	6.3	1.6	0.3	1.3	6.7	2.8	6.0–7.5	1.9–4.0	nd	
**16**	3-Br	Oct	H	H	6.9	9.7	8.0	0.0	0.6	0.3	11.2	40.6	10.3–12.1	29.5–56.1	nd	
**17**	3,4-diCl	m-MeOPhenethyl	H	H	6.9	8.6	6.6	1.3	0.1	0.6	>400	nd[c]			nd	

Table S1. *Cont.*

					IC$_{50}$ [a] (µM)			SD for IC$_{50}$ (µM)			BIC$_{50}$ [b] (µM)		95% Confidence Interval for BIC$_{50}$		*C.elegans*	
**n°**	**R4**	**R1**	**R2**	**R3**	**L1210**	**CEM**	**HeLa**	**L1210**	**CEM**	**HeLa**	***S.* Typhimurium**	***P. aeruginosa***	***S.* Typhimurium**	***P. aeruginosa***	**% Survival at 25 µM [d]**	**SD for % Survival at 25 µM**
			2N-substituted													
**18**	H	H	Bu	H	46.4	667.4	19.5	0.0	92.9	4.6	25.3	31.8	23.3–27.6	21.3–47.3	nd	
**19**	H	H	i-Bu	H	83.6	382.7	91.4	9.3	9.3	27.9	4.9	1.2	3.5–6.7	0.6 2.5	nd	
**20**	H	H	c-Pen	H	27.3	293.3	27.3	11.0	10.1	3.5	52.9	33.8	42.4–66.1	23.9–47.7	98.1	2.7
**21**	4-Cl	H	Bu	H	40.0	374.6	34.7	12.0	11.2	2.0	nd	nd			nd	
**22**	4-Cl	H	i-Bu	H	336.3	297.3	171.5	92.1	0.0	104.1	2	0.9	1.6–2.5	0.5–1.8	97.6	5.0
**23**	4-Cl	H	Pen	H	22.0	322.9	19.8	14.0	4.2	0.8	>400	~6.3			nd	
**24**	4-Cl	H	c-Pen	H	35.5	591.0	28.8	3.4	11.5	22.9	4.4	13.5	4.0–4.8	9.0–20.5	100.1	10.0
**25**	4-Br	H	Bu	H	125.8	493.0	50.7	6.0	10.0	5.2	7.1	9.8	3.7–13.9	6.8–14.1	102.4	5.7
**26**	4-Br	H	i-Bu	H	31.7	306.0	22.6	2.8	20.1	1.6	2.9	1.2	2.6–3.4	0.6–2.3	nd	
**27**	4-Br	H	Pen	H	14.9	375.2	16.3	10.4	14.0	1.6	3.1	10.2	2.0–5.0	2.9–35.9	nd	
**28**	4-Br	H	c-Pen	H	32.7	673.6	32.7	3.3	26.1	6.5	12.1	7.2	6.5–22.5	4.4–12.0	nd	
**29**	3,4-diCl	H	c-Pen	H	76.1	155.9	79.8	14.5	101.5	54.4	5.7	7.9	3.8–8.5	4.2–14.8	nd	
			4,5-disubstituted													
**30**	H	H	H	4-OMePh	64.1	52.8	45.2	3.8	3.8	15.1	77.1	nd	60.6–98.0		nd	
**31**	H	H	H	4-ClPh	59.3	48.2	40.8	7.4	0.0	7.4	46.9	nd	35.2 62.5		nd	
**32**	4-Cl	H	H	4-MePh	12.3	10.2	9.5	2.1	1.4	1.8	12.9	nd	10.7–15.6		nd	
**33**	4-Cl	H	H	4-CF_3Ph	41.5	35.5	26.1	3.0	3.0	2.1	10.8	nd	6.5–17.8		nd	
**34**	4-F	H	H	amidophenyl	74.2	74.2	77.6	3.4	23.6	6.7	182.1	nd	116.6–284.4		nd	
			1,4,5-trisubstituted													
**35**	4-Me	Ben	H	p-PenOBn	8.6	8.4	14.8	0.7	0.9	9.8	10.3	27.4	8.0–13.3	12.32–60.78	nd	
			2AI-triazole conjugates													
**36**	H	H	n = 2, R'= Ph	H	221.0	452.6	72.9	115.0	302.7	9.1	36.5	>400	22.9–58.1		87.7	11.2
**37**	H	H	n = 3, R' = Ph	H	217.8	459.2	106.7	101.6	290.4	52.3	40.0	19.0	32.3–49.5	8.8 to 41.0	86.4	0.1
**38**	4-OMe	H	n = 2, R' = 4-BrPh	H	18.4	596.6	16.8	0.2	4.6	6.8	91.2	42.7	40.4–205.9	22.4 to 81.7	nd	
**39**	4-Br	H	n = 2, R' = c-Hex	H	20.7	396.0	22.5	1.2	7.0	5.5	8.4	~12.5	6.7 to 10.4		99.3	6.5
**40**	4-Br	H	n = 2, R' = c-Pr	H	43.9	33.6	67.1	2.6	20.7	10.3	2.0	71.6	1.4 to 2.9	21.8 to 234.8	82.6	5.6

[a] IC50: compound concentration required to inhibit cell proliferation by 50%. The values represent the means of 3 repeats. Standard deviations (SD) are also provided.
[b] BIC50: compound concentration required to prevent biofilm formation (at 25 °C) by 50%, as previously reported [13–16]. 95% confidence intervals are also provided.
[c] nd: not determined. [d] The % survival of the worms in the presence of anti-biofilm compounds was calculated after 7 days relative to their viability at day 0. The values represent the means of least 2 repeats. Standard deviations (SD) are also provided.
